# Study of Wettability and Solderability of SiC Ceramics with Ni by Use of Sn-Sb-Ti Solder by Heating with Electron Beam in Vacuum

**DOI:** 10.3390/ma15155301

**Published:** 2022-08-01

**Authors:** Roman Kolenak, Igor Kostolny, Jaromir Drapala, Jan Urminsky, Alexej Pluhar, Paulina Babincova, Daniel Drimal

**Affiliations:** 1 Faculty of Materials Science and Technology in Trnava, Slovak University of Technology in Bratislava, Jána Bottu č. 2781/25, 917 24 Trnava, Slovakia; roman.kolenak@stuba.sk (R.K.); jan.urminsky@stuba.sk (J.U.); alexej.pluhar@stuba.sk (A.P.); paulina.babincova@stuba.sk (P.B.); 2 FMT—Faculty of Materials Science and Technology, Technical University of Ostrava, 17. Listopadu 15, 708 33 Ostrava, Czech Republic; jaromir.drapala@vsb.cz; 3 The First Welding Company Inc., Kopčianska 14, 851 01 Bratislava, Slovakia; drimal.daniel@pzvar.sk

**Keywords:** active solder, solderability, wettability, electron beam, SiC ceramics

## Abstract

The aim of this research was to study the wettability and solderability of SiC ceramics by the use of an active solder of the type Sn5Sb3Ti in a vacuum by electron beam heating. This solder exerts a narrow melting interval, and only one thermal effect, a peritectic reaction, was observed. The liquidus temperature of the solder is approximately 243 °C. The solder consists of a tin matrix where the Ti_6_(Sb,Sn)_5_ and TiSbSn phases are precipitated. The solder wettability on a SiC substrate decreases with decreasing soldering temperature. The best wetting angle of 33° was obtained in a vacuum at the temperature of 950 °C. The bond between the SiC ceramics and the solder was formed due to the interaction of Ti and Ni with silicon contained in the SiC ceramics. The formation of new TiSi_2_ and Ti_3_Ni_5_Si_6_ phases, which form the reaction layer and thus ensure the bond formation, was observed. The bond with Ni is formed due to the solubility of Ni in the tin solder. Two phases, namely the Ni_3_Sn_2_ and Ni_3_Sn phases, were identified in the transition zone of the Ni/Sn5Sb3Ti joint. The highest shear strength, around 40 MPa, was attained at the soldering temperature of 850 °C.

## 1. Introduction

Silicon carbide (SiC) is a ceramic used for temperatures up to around 1400 to 1600 °C. Its applications involve not only mechanical devices, such as mechanical joints or sliding bearings, but also electronic parts servicing at higher application temperatures [1]. In such cases it is necessary to join the SiC ceramics with different metals or metallic alloys.

The current acceptable technological solutions for the realization of SiC/metal joints face difficulties mainly due to different coefficients of thermal expansivity, which are often the reason for the failure of such joints [2]. Chen et al. [3] proposed the use of an interlayer with the desire to minimize the yield stress, and thereby maximize the potential for the relaxation of thermal expansion mismatch stresses by plastic flow. The SiC ceramic is a covalent material and almost non-wettable by pure metals [4]. 

The joining of ceramic materials in the electronic industry is mostly realized by the use of filler metals with lower contents of an active element (mostly Ti). Wei et al. [5] used titanium coating on Si_3_N_4_ ceramics to improve the brazing properties. Walker and Hodges [6] compared metal-ceramic brazing methods and pointed to the importance of the brazing alloy containing titanium for improving the wettability of ceramic materials. Xian [7] showed the importance of Ti in Sn solder for increasing the wettability of Si-Al-O-N ceramic. In further work, Qu et al. [8] pointed out the influence of the active elements Ti and Y in Sn-Ag-Ti-based solder for the soldering of dissimilar metals. They found out that the addition of a small amount of Y further improved the oxidation resistance because it could inhibit the oxidation of Ti in the molten solder and reduce the oxygen atoms entering into the solder. Titanium allows the wetting and joining of Ti, Al, Si, glass and different types of ceramics via the activation of soldered surfaces. Kolenak et al. [9] researched the solderability of AlN ceramics with Cu using a Sn-Ag-Ti solder. They found that Ti plays the major role in the AlN/solder bond formation. Ti is distributed to the interface with AlN ceramics owing to ultrasound assistance, where it creates a mutual reaction zone formed of the NTi and AlTi_2_ phases. Ultrasonic-assisted soldering was also studied in the work of Yan et al. [10] for joining aluminum metal matrix composites. The authors used a Zn-Al-based solder reinforced with SiC particles. With this filler metal, non-porous bonds strengthened with uniform particles were produced in the SiCp/A356 composite by ultrasonic vibration. Chaung et al. [11] investigated the zirconia brazing using AgCuTi and SnAgTi filler metals. The titanium in the filler metals segregated at the interface and formed a TiO reaction layer that was responsible for the wetting and bonding when a ZrO_2_ ceramic was brazed with Ag27Cu3Ti and Sn10Ag4Ti filler metals. In the case of soldering SiC, it is advisable to avoid the content of Al or Ag in the solder, since these metals form brittle silicides, thereby lowering the strength of fabricated joints [12].

The Sn-Ag-Ti alloy is the most used active solder base with a titanium addition, and was initially developed by S-Bond Technologies. This base is used to join the majority of metals, ceramics and composites. This solder was also used in the work of Cheng et al. [13] for soldering GaAs. Titanium (Ti) and gallium (Ga) were found to obviously take part in the active bonding between the GaAs substrate and the Sn3.5Ag4Ti(Ce, Ga) alloy filler. In addition, there was a resultant discontinuously formed along the interface, which was identified as Ga_4_Ti_5_. Azizi et al. [14] found that the Ti in the Sn3Ag4Ti alloy formed a 10 nm TiC layer at the graphite interface after the laser melting of metal structures onto a graphite substrate. The Sn-Ag-Ti-based solder can be applied without the flux or preliminary coating of substrates and is free from lead or cadmium, corresponding to the requirements of all the environmental initiatives for lead-free soldering (RoHS etc.) [15]. 

In spite of the fact that the manufacturer also claims the applicability of this solder for silicon carbide (SiC), its silver content slightly reduces the strength of the soldered joints. The development of an active solder is advancing, and other bases are being designed that may compete with S-Bond solders, which is why a new Sn-Sb-Ti-based solder was used in this work. The issue of designing new soldering alloys suitable for heavy-duty electronic equipment is divided by the type and purpose of the application. In some cases, it is necessary that the soldering alloy possesses a relatively low melting point. This is applicable in the case of so-called gradual soldering, where during the assembly of the packaged part some solder must have a lower melting point than the others. This solution is necessary in order to avoid damaging the already soldered joints in the assembled overall part [16]. For solving such a case, the solders based on Sn-Cu, Sn-In, Sn-Bi [17] or Bi-Sn have been applied [18]. 

The opposite case consists of designing a suitable alloy that must resist higher application temperatures in the part [19]. The solders suitable for higher application temperatures should possess a melting point in the approximate range from 260 °C to 420 °C [20]. For this purpose, the Sn-Sb-Ti solder was applied for soldering the combination of SiC/Ni joints. 

Since the considered solder is an active one, this combination can be soldered by a direct flux-free process. However, the application of suitable technology that allows the direct flux-free soldering is essential. At present, several possibilities for fabricating such joints are documented. Great attention is mainly devoted to ultrasonic flux-free soldering. Ultrasonic waves cannot only disrupt the natural oxide layers on the surface of molten metal but also strengthen the bond boundary by supporting the interfacial reaction. Wu et al. [21] investigated the microstructural evolution of SiC joints that were soldered using Zn-Al filler metals with the assistance of ultrasound. Their other works [22] studied these joints using Sn-Zn-Al and Sn-Ag-Ti solder alloys [23]. In their works, the authors claim that ultrasonic cavitation-induced heterogeneous nucleation was the refinement mechanism of the bond layer of the joints, and that ultrasonic action improved the shear strength of these joints. Ultrasonic-induced capillary action and cavitation was also the main mechanism of bond formation in the work of Xu et al. [24]. The method of a direct ultrasonic soldering of a ceramic Al_2_O_3_ substrate with copper was dealt with in the study of Hasan et al. [25]. The joint was fabricated by the use of a Zn14Al solder. High-quality joints without visible defects were attained at optimized parameters. The highest shear strength of the Al_2_O_3_/Zn14Al/Cu joint, namely 80 MPa, was achieved at the soldering temperature of 480 °C, the ultrasound power of 200 W and the soldering time of 30 s. Tschudin et al. [26] dealt with the ultrasonic soldering of SiC ceramics. In their study, they used the solder type Zn8.5Al1Mg. Soldering was performed at the temperature of 420 °C. They found that the strength of the joints increased by prolonging the time of ultrasonic activation. The authors obtained the highest shear strength of 148.1 MPa at the time of ultrasonic activation of 8 s. A new amorphous layer from 2 to 6 nm in thickness was formed on the boundary between the solder and substrate. However, it is technologically demanding to fabricate large-area joints by ultrasonic soldering. Therefore, such joints are fabricated in a vacuum with the precise positioning of the soldered materials and the solder inserted between them. This technology, employed by the use of active solders with Ti content, is used for soldering ceramic materials at high temperatures above 700 °C due to the necessity of thermodynamic activation. In our previous study [27], we confirmed the fact that the wettability of the Sn3.5Ag4Ti(Ce, Ga) solder depends on the temperature and time of wetting. The wettability of Sn3.5Ag4Ti(Ce, Ga) on Al_2_O_3_ was attained by heating at 850 °C for 43 min. Chai et al. [28] investigated the wettability of the Sn10Ag4Ti solder on SiC and Al_2_O_3_ substrates. They stated that the contact angles decreased with increasing temperature and time of heating. The contact angle of the Sn10Ag4Ti solder on SiC decreased below 15° when the temperature was raised above 680 °C. As reported by Beata Synkiewicz et al. [29], the use of the vacuum unit resulted in a drastic decrease in both the number of voids in the joints and their total area in the solders. The vacuum generated by the system allows the easier suction of excess gas from the interior parts of solder joints (smaller opposing forces have to be overcome) and the pressure impedes the release of voids outside of them. The influence of a vacuum on the quality of soldered joints was also supported by the authors’ studies [30,31,32,33]. Their results showed a significant reduction in voids in the solder joint. Based on the achieved results, they stated that soldering using a high vacuum can reduce the incidence of defects by up to 5% and can stabilize the size of the intermetallic phases in the range of 1 to 3 μm. These factors have a major impact on the strength and reliability of soldered joints.

Long soldering times in a vacuum may be reduced by a rapid heating process. Soldering with an electron beam in a vacuum can be suitable for this purpose. However, this method of soldering with active solders is not often mentioned in the literature. Therefore, the experiments with soldering ceramics with an active solder of the type Sn5Sb3Ti were performed under the conditions of high-temperature activation with an electron beam in a vacuum at temperatures ranging from 750 to 950 °C. A high soldering temperature (above 700 °C) is necessary for titanium activation in the solder. The aim of the experiments was to assess the effect of soldering temperature on solder wettability on an SiC substrate, to identify the reaction products in the joint boundary, and to determine the effect of soldering temperature on the shear strength of the joints.

## 2. Materials and Methods

The manufacture of experimental soldering alloy in the as-cast condition was realized in a vacuum oven at argon overpressure of (4.6) 200 Mbar. As the input components for solder manufacturing, the materials with the high purity of 4N were used. The temperature of the solder melting was around 1100 °C. Titanium was slowly dissolved in the solder. 

The chemical composition of the prepared soldering alloy is given in Table 1. The chemical analysis of alloys was performed by atomic emission spectrometry with induction coupled plasma (ICP-AES). The analysis was realized on the equipment type SPECTRO VISION EOP. 

The analysis proper was performed on the emission spectrometer with a pneumatic atomizer and Scott’s sputtering chamber.

The experiments were performed with the solders and substrates of the following materials and forms:

- solder type Sn5Sb3Ti in the form of a cube with dimensions 4 mm × 4 mm × 4 mm, applied for wettability measurement.
- solder type Sn5Sb3Ti in the form of a foil with dimensions Ø 15 mm × 0.2 mm for preparation of specimens for the analysis of boundaries in soldered joints.
- solder type Sn5Sb3Ti in the form of a foil with dimensions 10 mm × 10 mm × 0.2 mm for preparation of specimens for shear strength measurement.
- ceramic substrate type SiC in the form of discs with dimensions Ø 15 mm × 3 mm and for the shear strength test in the form of squares with dimensions 10 mm × 10 mm × 3 mm.
- nickel substrate in the form of discs with Ø 15 mm × 3 mm.

**Figure 1:**
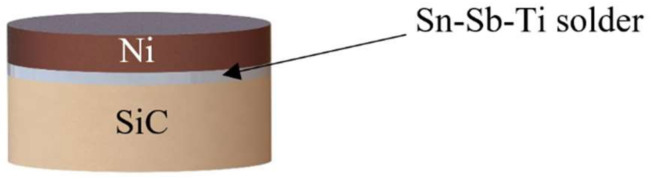
Assembly of a soldered joint for the analysis of solder/substrate [34].

The scheme of the soldered joint prepared for the chemical analysis of solder/substrate boundaries is shown in Figure 1.

**Figure 2:**
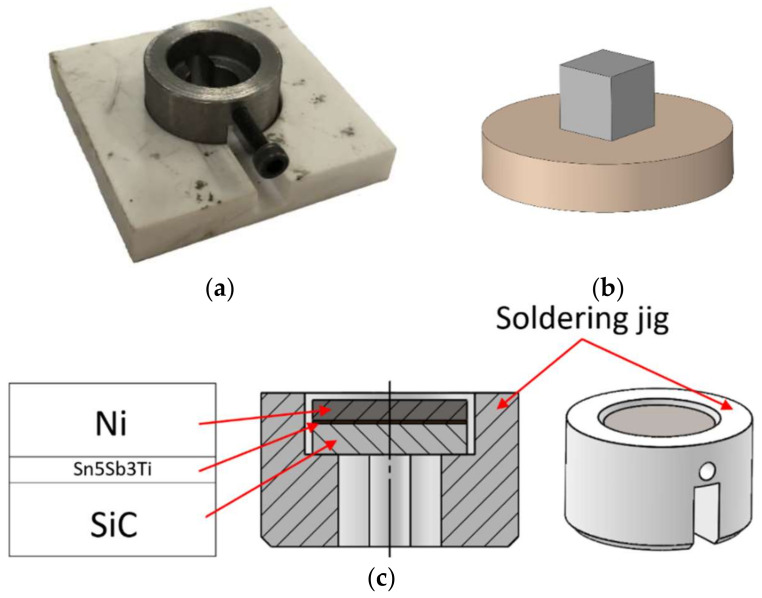
Graphical representation of a jig for experimental soldering. (**a**) real representation; (**b**) layout of solder for shear strength measurement; (**c**) schematic, in section representation of layout of soldered assembly in the jig.

The joints were fabricated by electron beam soldering in a vacuum. The heating of the soldered assembly was ensured via heating of a special metallic jig in which the experimental assembly was inserted (Figure 2a). The impinging electron beam was defocused to the shape of a circle in order that the emitted electrons would only impinge on the metallic jig, which was thus heated, but not on the specimen proper. The specimen was thus heated by the radiant and conductive heat at the temperatures of 750, 850, or 950 °C. The temperature was monitored by a set of type K thermocouples. 

The wettability of the solder type Sn5Sb3Ti was analyzed by the precise setting of the solder in the form of a cube on a circular SiC substrate. It was measured and compared at all temperature levels. The specimen for the wettability measurement is shown in Figure 2b. A similar jig was also used for the layout of the soldering assembly for the fabrication of the soldered joint of SiC/Ni (Figure 2c). A foil of Sn5Sb3Ti solder, 0.2 mm in thickness, was inserted between the soldered materials.

**Figure 3:**
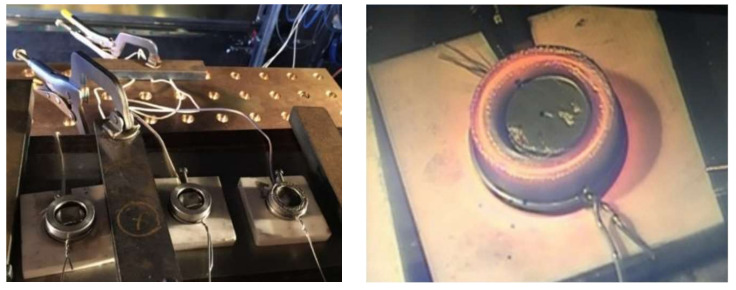
Setting the jig with specimens on the working table and real representation of the soldering process.

After the proper setting of the jig with the specimens on the working table (Figure 3), soldering was performed based on the parameters given in Table 2.

The soldering process necessitated the oscillation of the electron beam (see Figure 3) in order to attain uniform heating of the jig. The oscillation with a circular shape was thus necessary. The shape of the oscillation was defined by the following parametric equation:

(1) $$\left[\begin{array}{c} x\left(\theta \right)\\ y\left(\theta \right) \end{array}\right]=\left[\begin{array}{c} a+r\cos\theta \\ b+r\sin\theta \end{array}\right],\;<0;2\pi >$$

The magnitudes of oscillation were selected with respect to the shape of the jig and the distance of the jig surface from the electron cannon orifice. The parameters of oscillation are given in Table 3.

The specimens of the solder and soldered joints were prepared by the standard metallographic methods of grinding, polishing and etching. 

The solder and soldered joint microstructures were studied by the use of scanning electron microscopy (SEM) on microscope types TESCAN VEGA 3 and JEOL 7600 F (Tescan Orsay Holding, Brno, Czech Republic) with an X-ray micro-analyzer type Microspec WDX-3PC (Microspec Ltd., Peterborough, NH, USA) for performing the qualitative and semi-quantitative chemical analysis. 

The phase composition of the solder was identified by X-ray diffraction analysis. It was performed on solder specimens with dimensions of 10 mm × 10 mm on an XRD diffractometer type PANalytical X’Pert PRO (Malvern Panalytical Ltd., Malvern, UK).

The specimens prepared for the wettability measurement were crosscut and metallographically prepared for the wettability assessment of the solder on the SiC substrate. The measurement of the wetting angles at the given temperatures was performed by the goniometric method on macro cross sections of the specimens. 

**Figure 4:**
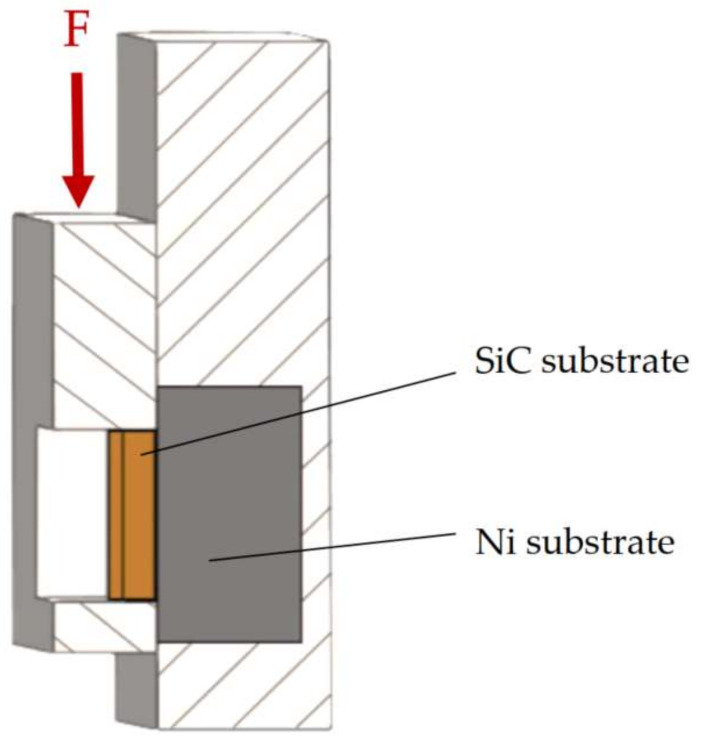
Scheme of shear strength measurement [34].

For the determination of the mechanical properties, the shear strength test was performed. The shear strength was measured on a versatile tearing equipment type LabTest 5.250SP1-VM (Labortech Ltd., Praque, Czech Republic). To change the direction of the axial tensile loading force, a jig with a defined shape of the test specimen was used (Figure 4). The shearing jig ensures a uniform loading of the specimen by shearing in the plane of the solder and substrate boundary.

## 3. Results

### 3.1. DTA Analysis

The DTA analysis of the studied alloy was performed. The analysis was always performed twice at the heating and cooling rate of 5 °C/min. According to Chen et al. [35], the following peritectic reaction takes place in the binary system of Sn-Sb:

L + Sn_3_Sb_2_ = (Sn) (2)

Based on our previous research [34], the onset point at double heating corresponded to temperatures of 227.8 °C and 225.9 °C, while during cooling it corresponded to temperatures of 224.6 °C and 224.1 °C. The peak at heating corresponded to temperatures of 240.2 and 241.6 °C. The DTA analysis did not reveal any other reactions. The difference in the onset point values at heating was caused by the heterogeneity of the initial alloy due to precipitated phases, namely the primary solidified phase with an acicular morphology and a high titanium content, i.e., Ti_6_(Sn,Sb)_5_, whereby a considerable portion of titanium was exhausted, and the remaining titanium reacted with the Sn-Sb melt, forming a brittle TiSnSb phase [34].

### 3.2. Microstructure of Sn5Sb3Ti Solder

**Figure 5:**
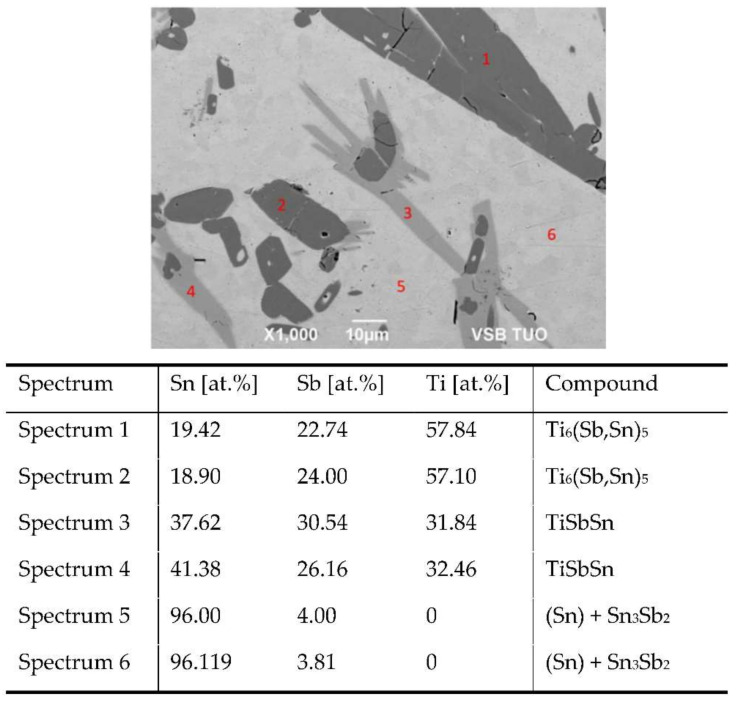
Point EDX analysis of Sn5Sb3Ti solder.

The solder structure, which was also analyzed in our previous study [34], consists of a tin matrix, where irregular constituents of intermetallic phases of tin and antimony occur. In principle, two kinds of phases, namely the light-grey and dark-grey, are of concern. Some constituents have an outer light-grey zone, which is in contact with tin. Inside them, a dark-grey zone was observed. To determine the chemical composition in individual components of the soldering alloy, the EDX analysis was performed. The points of measurement are shown in Figure 5 and are marked with numerals from 1 to 6.

For the points of measurement 1 and 2, the dark-grey phase, the stoichiometry corresponds to the composition of the intermetallic compound Ti_6_(Sb,Sn)_5_. It is relatively brittle, with an HV hardness of 0.01–939. Tin and antimony in the Ti_6_(Sb,Sn)_5_ phase are mutually substituting each other. 

For the points of measurement 3 and 4, the light-grey phase mostly occurs inside the matrix of the Sn(Sb) solder, but less frequently on the surface of the Ti_6_(Sb,Sn)_5_ phase. It is in direct contact with the solder matrix. This phase corresponds to the stoichiometric composition of the TiSbSn compound. Its hardness is HV 0.01–174. 

For the points of measurement 5 and 6, the solder matrix consists of a solid solution (Sn). In the metallographic image (Figure 5), a minority of tiny constituents of the Sn_3_Sb_2_ phase may be observed. Titanium did not occur in this location.

**Figure 6:**
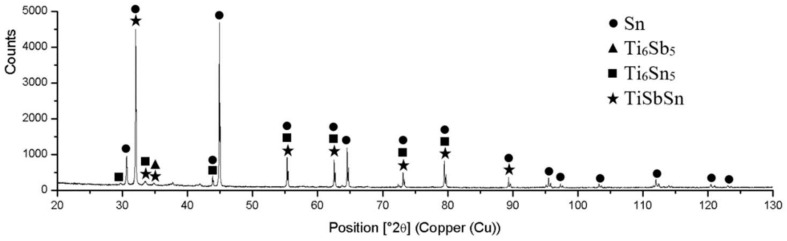
XRD analysis of Sn5Sb3Tisolder [34].

The diffraction XRD analysis of Sn5Sb3Ti was analyzed in our previous study [34]. The solder proved the presence of tin (Sn) and antimony (Sb), as well as the presence of intermetallic phases of titanium and antimony, namely Ti_6_Sb_5_, Ti_6_Sn_5_ and TiSbSn. The record from the diffraction analysis is shown in Figure 6.

### 3.3. Wettability and Interaction of Sn5Sb3Ti Solder on the Surface of SiC Ceramics

**Figure 7:**
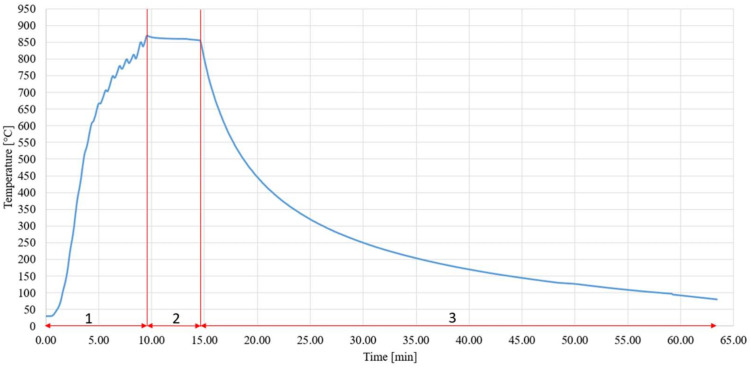
Thermal cycle of soldering with following thermal phases: 1—heating phase, the rate of 90 °C/min; 2—holding phase, the average temperature of 853 °C during 5 min; 3—cooling phase, the rate of 15.6 °C/min.

The wettability test was performed in a vacuum, while heating was realized via an electron beam. Regarding the fact that SiC ceramic is non-conductive and therefore cannot be heated by the impinging electrons, a metallic jig (shown in Figure 2a) was applied. One great advantage of soldering with an electron beam is the fact that the soldering process takes place in a vacuum, which is essential in the case of soldering with active solders that contain an active element with a high affinity for oxygen. Another advantage is offered by a shorter work cycle of the soldering than in the case of heating in a vacuum oven, since it is performed by a highly concentrated heat source. An example of the work cycle of soldering in the case of the wettability measurement at the temperature of 850 °C is shown in Figure 7. This cycle consists of a rapid heating rate at the soldering temperature of approximately 90 °C/min. The dwell time at the soldering temperature was approximately 5 min, followed by a slow cooling at approximately 15.6 °C/min. The experiments were performed at the temperatures of 750, 850 and 950 °C. The temperature of 750 °C was proposed on the basis of a previous study [27], where the wettability of Al_2_O_3_ ceramics by a Sn-based solder containing 2 wt.% Ti was studied. This study showed that the high-temperature activation of an active element in the solder (Ti) necessitated a minimum temperature of 750 °C.

**Figure 8:**
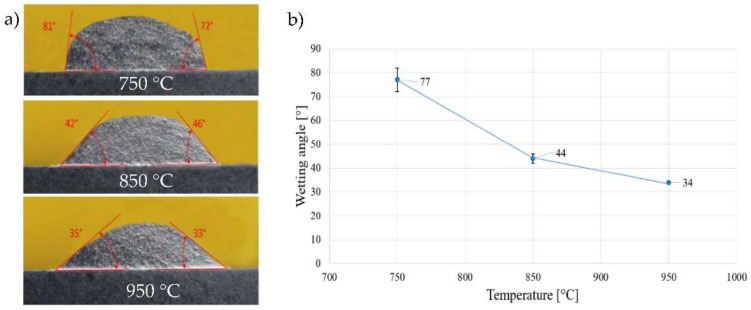
Wettability of SiC ceramics by Sn5Sb3Ti solder at the temperatures of 750, 850 and 950 °C. (**a**) wetting angles; (**b**) course of wettability measurement.

The aim of the experiment was to determine the wettability of an active solder of the type Sn5Sb3Ti on the surface of ceramic material of SiC. The course of wettability is shown in Figure 8. From the results of the wettability course, it is obvious that with increasing soldering temperature the wetting angle also decreased. At the temperature of 750 °C, the average wetting angle attained approximately 77°. With the temperature increase to 850 °C, the wetting angle decreased to the average value of 44°. The lowest wetting angle was achieved at the temperature of 950 °C, when it attained an average value around 33°. From the results of the wettability measurements, it is obvious that the parameters were properly designed.

To identify the interactions between the solder type Sn5Sb3Ti and SiC ceramics, a specimen prepared at the temperature of 850 °C was selected from among all of the specimens prepared for the wettability test. In the case of the specimen prepared at the temperature of 750 °C, sufficient wettability (77°) is not ensured. However, the temperature of 950 °C is already too high for the fabrication of joints with Ni, and the solder particles start to partially evaporate. 

**Figure 9:**
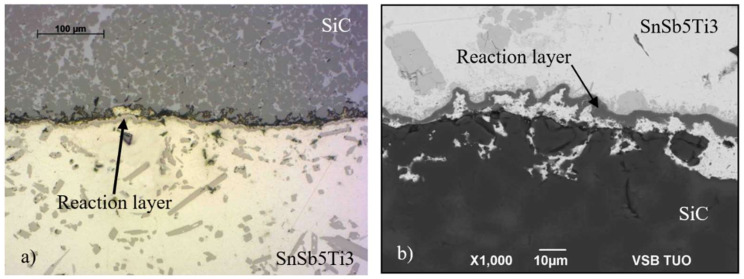
Microstructure on SiC/Sn5Sb3Ti joint boundary of wettability specimen prepared at the temperature of 850 °C (**a**) from the optical microscope; (**b**) from the SEM analysis.

**Figure 10:**
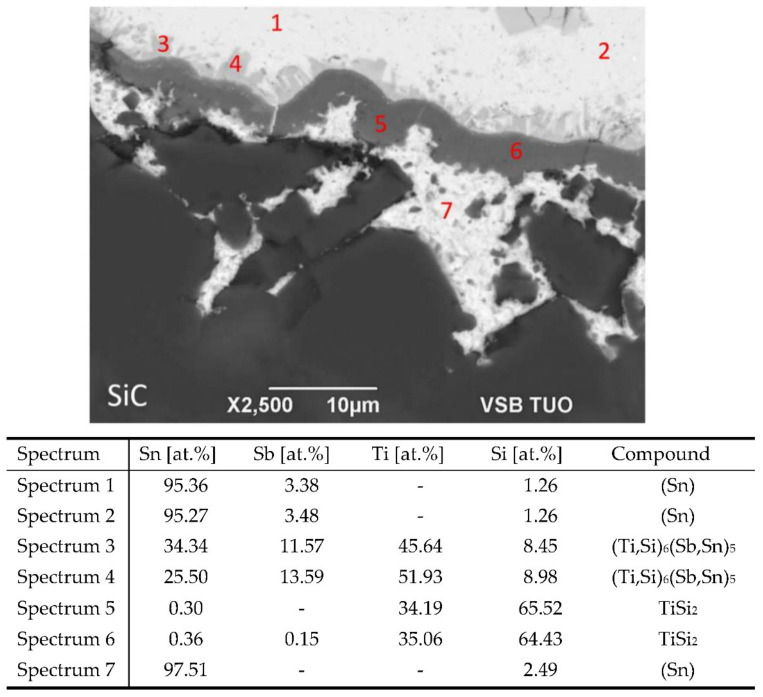
Point EDX analysis of solder from SiC/Sn5Sb3Ti joint boundary of the specimen for wettability test.

The microstructure of the joint boundary from the optical analysis of the SiC/solder is documented in Figure 9. The interaction and formation of a reaction layer may be observed on the ceramic/solder boundary. For the determination of the chemical composition of the solder and the new-formed phases, the EDX analysis was applied. Figure 10 shows the points of measurement where the analysis of chemical composition was performed.

From the results of the EDX analysis, the following findings may be concluded: 

The points of measurement 1 and 2 revealed bright zones in the vicinity of the solder/SiC ceramic boundary. It concerns the solid solution (Sn) with dissolved Sb and Si elements at the level of 3.48 at.% Sb and 1.26 at.% Si. The points of measurement 3 and 4 represent the phase that is connected with the reaction layer. It consists of Ti_6_(Sb,Sn)_5_, where part of the titanium was substituted with silicon with an approximate concentration of 8.5 at.% Si. The interaction between the ceramic and solder was thus definitively proved. The points of measurement 5 and 6 were directly in the reaction layer on the solder/SiC ceramic boundary. This unambiguously concerns the TiSi_2_ phase, which formed due to the interaction of the solder and the SiC ceramic.

In the point of measurement 7, between the reaction layer and the SiC ceramic, a solid solution (Sn) was identified with dissolved Si at the level of 2.49 at.%.

A sound bond was formed in the SiC/solder boundary owing to the interaction of the solder with SiC, where in the boundary vicinity a reaction layer was identified that was formed from the TiSi_2_ phase and the (Ti,Si)_6_(Sb,Sn)_5_ phase interlocked with the reaction layer.

The mechanism of bond formation in the wettability test is as follows. During the soldering process performed at the temperature of 850 °C, the Ti contained in the solder bulk is distributed by the mechanism of diffusion to the ceramic/solder boundary, where it primarily reacts with the silicon in the SiC ceramic to form a reaction layer composed of a new phase, which is formed according to the following formula:

/Ti/ + 2Si → TiSi_2_ (3)

The intergranular spaces of the SiC ceramics, where the silicon was initially infiltrated, are subsequently filled with a tin matrix with partially dissolved silicon. Thus, behind the reaction layer a new composite is formed, composed of a tin matrix where the SiC grains are distributed.

### 3.4. Microstructure of SiC/Sn5Sb3Ti/Ni Joint

**Figure 11:**
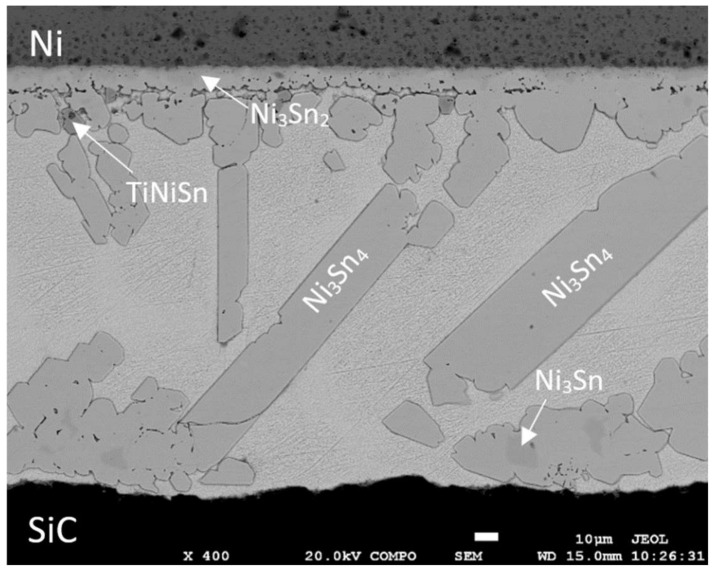
Microstructure of SiC/Sn5Sb3Ti/Ni joint from SEM analysis performed in BSE mode.

The fabrication of the SiC/Sn5Sb3Ti/Ni joint was performed on the basis of the results from the wettability test. The soldering temperature of 850 °C was selected, at which the average wetting angle of 44° was attained. Soldering was realized in a vacuum chamber with the thermal cycle shown in Figure 7. Owing to the high-temperature activation of an active element (Ti), an acceptable joint was achieved by the soldering process, free from any cracks or inhomogeneities. The microstructure of the soldered joint is shown in Figure 11.

From Figure 11 it is obvious that large constituents of the Ni-Ni_3_Sn_4_ phase occurred in the solder matrix after soldering. In the case of soldering at the temperature of 850 °C, a massive dissolving of Ni in the tin matrix occurred. On the boundary with nickel, a continuous transition zone was formed with the formation of new intermetallic phases. 

**Figure 12:**
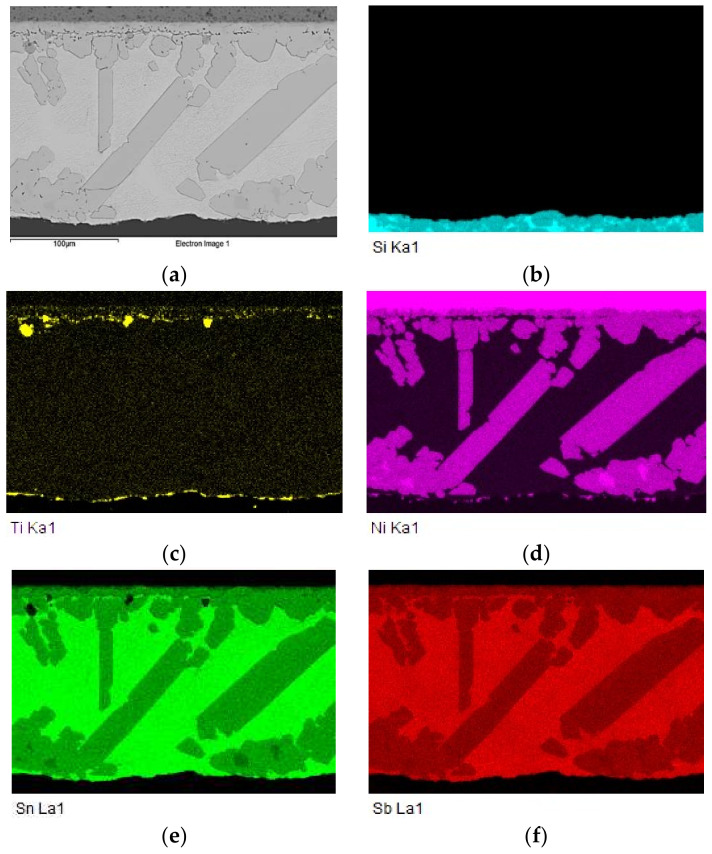
Planar distribution of Si, Ti, Ni, Sn and Sb elements on the boundary of SiC/Sn5Sb3Ti/Ni joint; (**a**) joint microstructure; (**b**) Si; (**c**) Ti; (**d**) Ni; (**e**) Sn; (**f**) Sb.

The map of elements in the SiC/Sn5Sb3Ti/Ni joint boundary in Figure 12 shows that titanium (Figure 12c) was distributed by the diffusion mechanism from the entire solder volume to the boundary with the SiC ceramic, but also partially to the boundary with Ni. Thus, the titanium phases such as Ti_6_(Sb,Sn)_5_ and TiSbSn, from the initial solder microstructure shown in Figure 5, disappeared. The map of the Ni element (Figure 12d) shows that nickel was partially segregated on the boundary with the SiC ceramic.

### 3.5. Analysis of Transition Zone in SiC/Sn5Sb3Ti Joint

Based on the results of the analysis of specimens from the wettability test, it was supposed that the active Ti element became concentrated on the boundary with the ceramic material (SiC), where it formed the reaction layer consisting mainly of the TiSi_2_ phase. 

**Figure 13:**
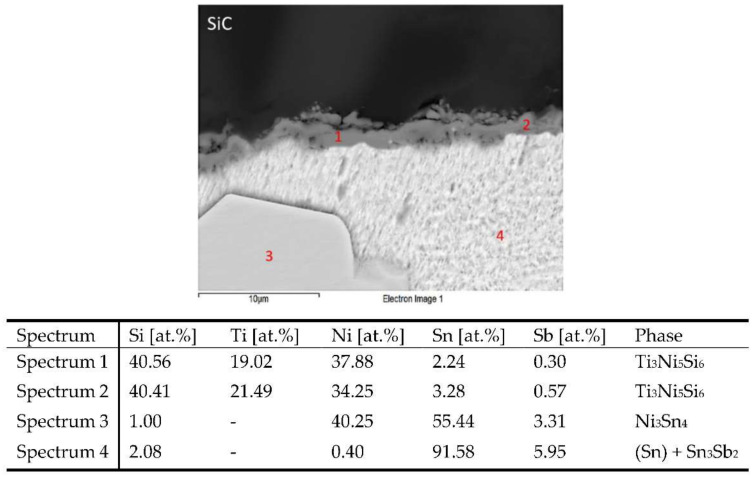
Point EDX analysis of SiC/Sn5Sb3Ti joint boundary.

The microstructure of SiC/Sn5Sb3Ti joint boundary is shown in Figure 13. The interaction of titanium was observed on the solder/SiC ceramic boundary with the formation of a reaction layer, whereby a greater amount of Ti segregated to this boundary. The point analysis proved the presence of titanium in amounts from 19 to 21.49 at.%. However, in the reaction layer on the boundary, the presence of an increased amount of Ni from 34.25 to 37.88 wt.% was also observed. It was found that both of these elements contribute to the bond formation with the ceramic material of SiC. The presence of silicon in the reaction layer in the amount around 40 at.% is proof of the mutual interaction between the solder and the ceramic substrate.

In contrary to the SiC/Sn5Sb3Ti joint boundary observed in the wettability specimen shown in Figure 10, in the case of SiC/Sn5Sb3Ti joint boundary with Ni, the presence of the TiSi_2_ phase in the reaction layer was not observed. The composition of the reaction layer was modified by nickel dissolved in the tin matrix of the solder. The reaction layer was formed of a new phase, namely Ti_3_Ni_5_Si_6_. According to Weitzer et al. [36], the Ti_3_Ni_5_Si_6_ phase lies between the intermetallic phases τ4 (TiNiSi) and τ7 (Ti_14_Ni_49_Si_37_).

**Figure 14:**
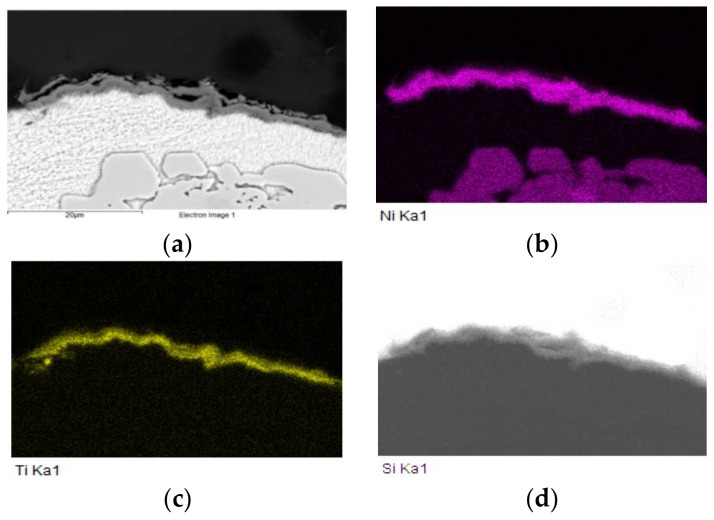

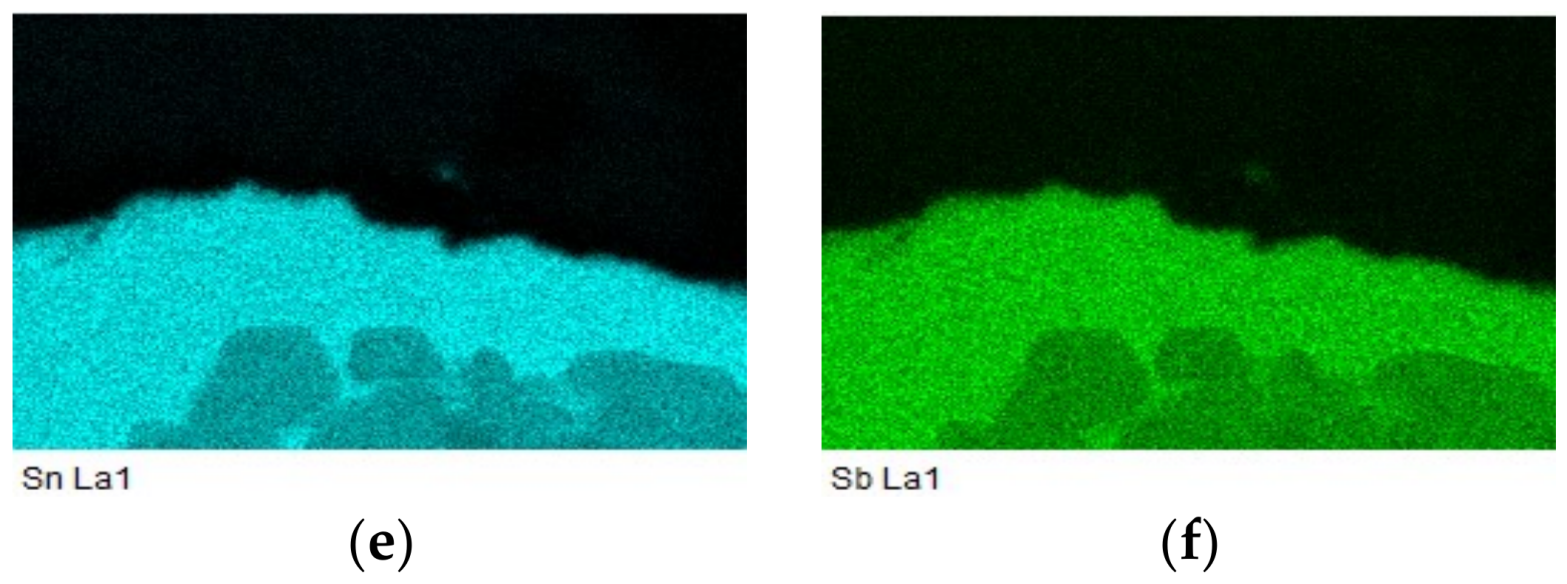
Planar distribution of Ni, Ti, Si, Sn and Sb elements on the boundary of SiC/Sn5Sb3Ti joint; (**a**) boundary; (**b**) Ni; (**c**) Ti; (**d**) Si; (**e**) Sn; (**f**) Sb.

The distribution of the reaction layer on the boundary of SiC/Sn5Sb3Ti joint formed of the new Ti_3_Ni_5_Si_6_ phase, containing Ti, Ni and Si, is well seen in the map of the Ti (Figure 14c) and Ni (Figure 14b) elements.

**Figure 15:**
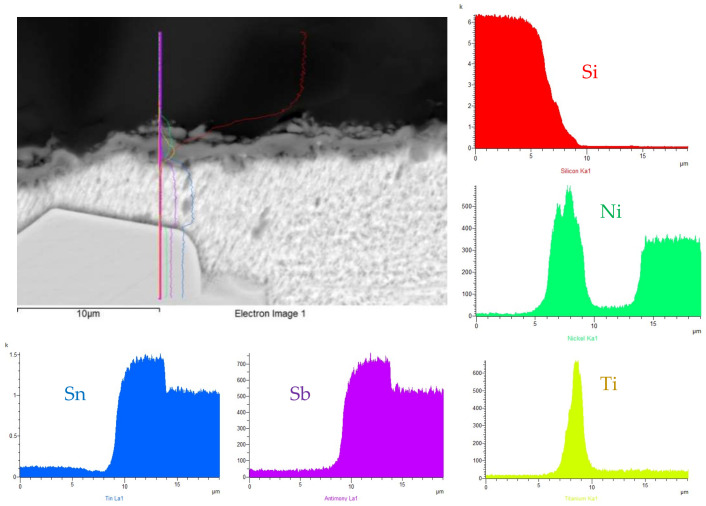
Line EDX analysis of SiC/Sn5Sb3Ti joint transition zone with a marked line and concentration profiles of Si, Ti, Ni, Sn and Sb elements.

The line analysis and concentration profiles of the Ti and Ni elements (Figure 15) prove that both of these elements were segregated on the boundary with the ceramic material of SiC.

From the mentioned facts, the following mechanism of bond formation may be concluded. Titanium was distributed during the soldering process from the solder to the boundary with the ceramic material (SiC) by the mechanism of diffusion. Nickel was dissolved in the tin solder and was also distributed to the boundary with the ceramic material by the mechanism of diffusion, where a reaction layer, ensuring the wetting of the SiC ceramic, was formed. Between Ti, Ni and the ceramic material, a reaction took place with the formation of reaction products, which allowed the wetting of the ceramics by an active solder. The thickness of the reaction layer was from 2 to 5 µm.

### 3.6. Analysis of Transition Zone in Ni/Sn5Sb3Ti Joint

In the boundary of the Ni/Sn5Sb3Ti joint, a uniform pronounced transition zone was formed where the phases of Ni and Sn were observed. The transition zone containing new phases was formed due to the dissolution of nickel in the molten tin solder at elevated temperature. In the work of Massalski [37], nickel started to dissolve in the tin solder at the temperature around 430 °C. The thickness of the new transition zone with intermetallic phases of nickel was from 9 to 18 µm.

**Figure 16:**
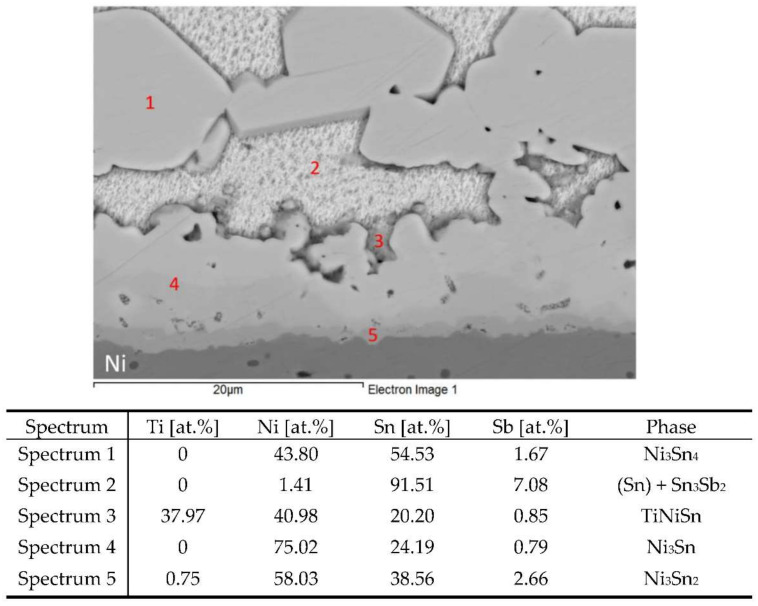
EDX point analysis of boundary in Ni/Sn5Sb3Ti joint.

The measurement of the chemical composition in this zone and adjacent regions was performed by EDX analysis at five points (spectra). The results of the measurement are given in the Table below Figure 16. 

The results of the point EDX analysis of the transition zone in the Ni/Sn5Sb3Ti joint proved that an intense interaction between nickel and the solder took place. Especially pronounced was the Ni_3_Sn_4_ phase (Spectrum 1), which penetrated the solder in the form of long tabular constituents and occupied a relatively large volume in the solder. In the boundary of the Ni/Sn5Sb3Ti joint, a thin layer of a stable Ni_3_Sn (Spectrum 4) phase, rich in nickel, was formed. Behind it, a thicker layer of Ni_3_Sn_2_ (Spectrum 5) phase followed. This phase was also identified as the scarce islands of the Ni_3_Sn_4_ phase at a larger distance from the boundary in the solder volume. Thus, all of the phases were identified in agreement with the binary diagram of Ni-Sn [37].

Titanium in the vicinity of the boundary in the Ni/Sn5Sb3Ti joint was localized in small zones as a dark phase in the SEM images. Titanium reacted with both nickel and the solder to form TiNiSn (Spectrum 3 phase). This phase exerted a strengthening effect on the mechanical properties of the soldered joint.

**Figure 17:**
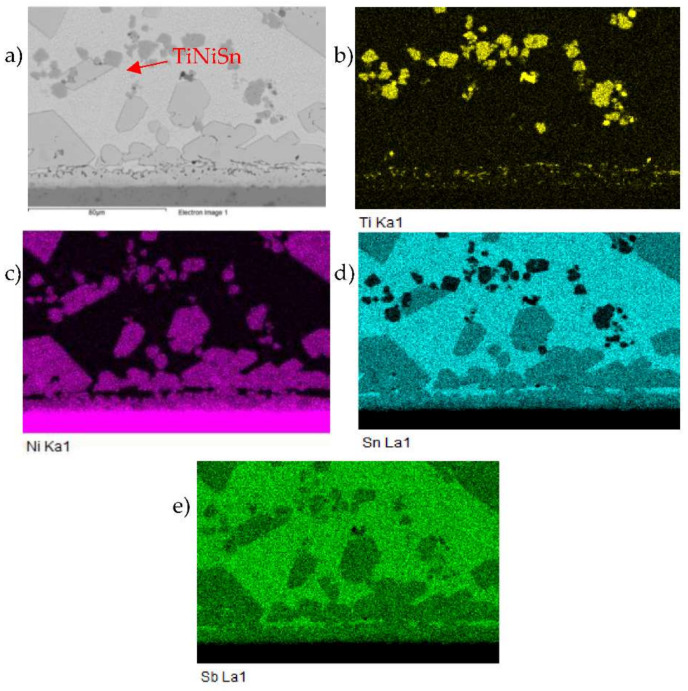
Map of elements in the boundary of Ni/Sn5Sb3Ti joint; (**a**) joint boundary; (**b**) Ti; (**c**) Ni; (**d**) Sn; (**e**) Sb.

The map of the distribution of the Ti, Ni and Sn elements on the boundary of the Ni/Sn5Sb3Ti joint is shown in Figure 17. Figure 17a,b show a clearly observable titanium phase in the matrix of the Sn5Sb3Ti solder.

**Figure 18:**
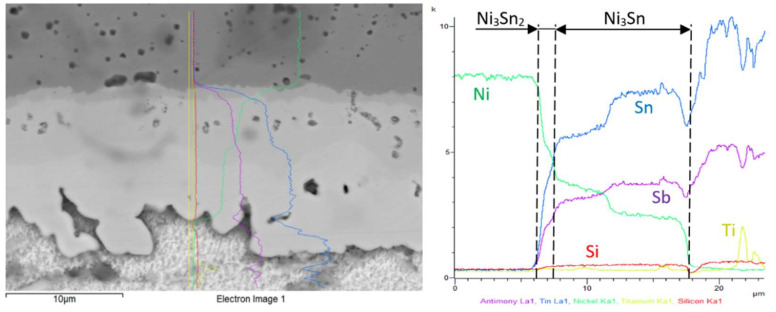
Concentration profiles of Ni, Sn, Sb and Ti elements in the boundary of Ni/Sn5Sb3Ti joint.

The concentration profiles of the Ni, Sn, Sb and Ti elements (Figure 18) in the boundary of the Ni/Sn5Sb3Ti joint revealed the occurrence of zones with the following nickel phases: Ni_3_Sn_2_ and Ni_3_Sn. The zone with the Ni_3_Sn_2_ phase was 1 µm in thickness and the zone with Ni_3_Sn phase was around 10 µm thick at the measured point.

**Figure 19:**
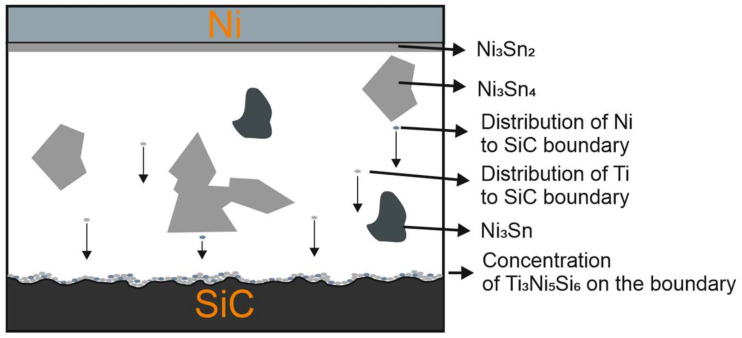
Mechanism of bond formation with Sn5Sb3Ti solder.

Based on the achieved results and observations, a graphic model of the mechanism of the bond formation between SiC and Ni was constructed (Figure 19).

### 3.7. Shear Strength of Soldered Joints

**Figure 20:**
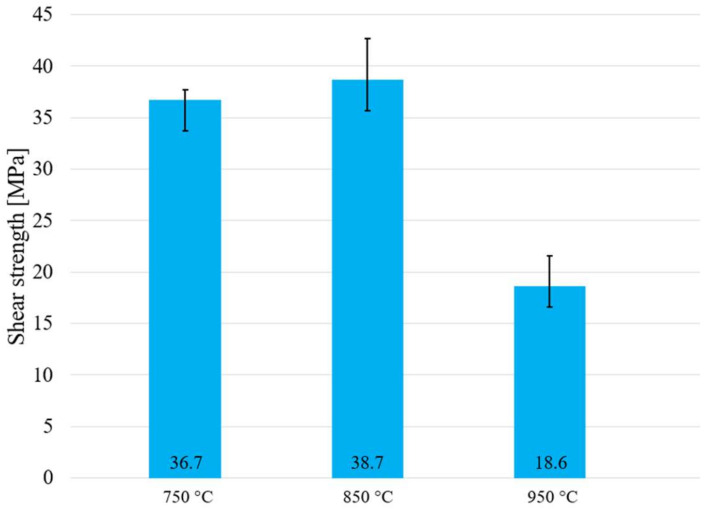
Shear strength of soldered joint of SiC/Ni fabricated with solder type Sn5Sb3Ti and dependence on soldering temperature.

The research in this study was oriented to the high-temperature soldering of SiC ceramics with a Ni substrate. Due to the expected potential of the active solder of the type Sn5Sb3Ti and its further application in practice, the testing of shear strength was performed at the temperatures of 750, 850 and 950 °C. The measurement was performed on three specimens of each material. The results of the average shear strength are shown in Figure 20. The highest average shear strength in the combination of ceramics/metal was attained at the temperature of 850 °C, as expected. In the case of the temperature of 850 °C, acceptable wettability was achieved with the wetting angle of 44° and the solder components had not yet evaporated. The wetting angle of 33° at the temperature of 750 °C was lower than at the temperature of 850 °C, so a lower shear strength may be supposed. The lowest shear strength was obtained at 950 °C, where the evaporation of the solder components, mainly Sn, occurred [38], and thus a lower shear strength of the joint was attained. 

**Figure 21:**
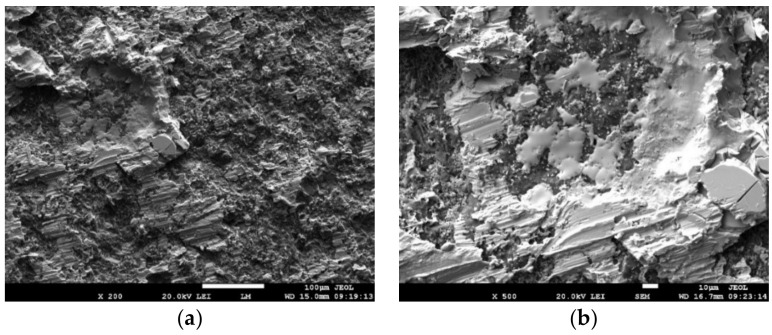
Fractured surface of soldered joint of SiC/Sn5Sb3Ti/Ni; (**a**) magnification 200×; (**b**) magnification 500×.

**Figure 22:**
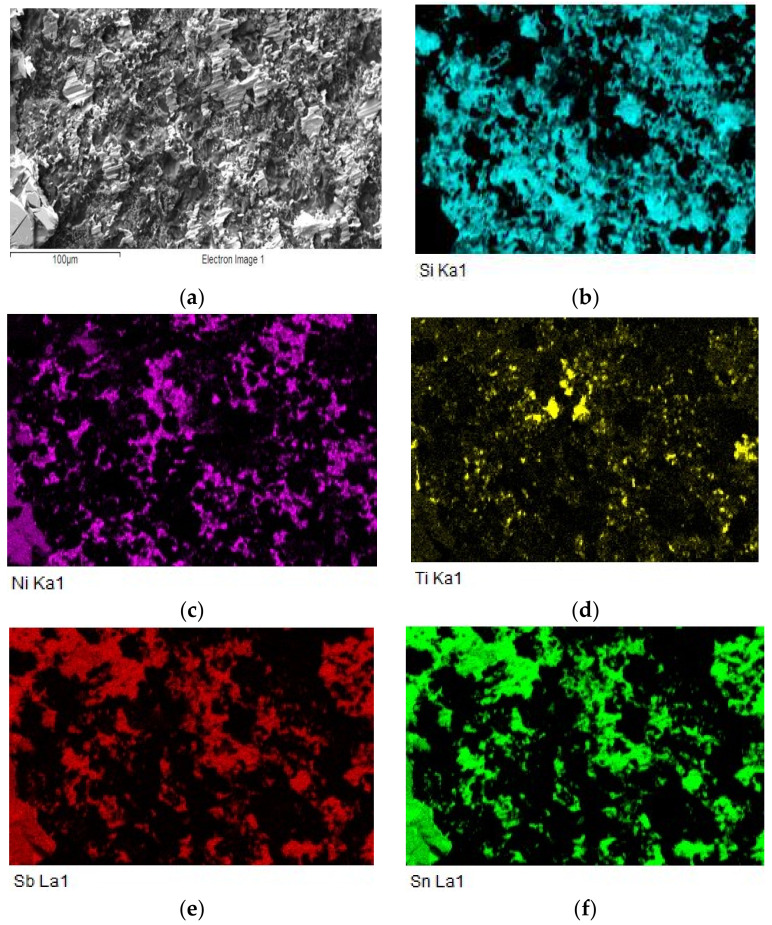
Fractured surface of soldered joint of SiC/Sn5Sb3Ti/Ni and the planar distribution of individual elements; (**a**) fracture morphology; (**b**) Si; (**c**) Ni; (**d**) Ti; (**e**) Sb; (**f**) Sn.

For a more exact identification of the mechanism of bond formation, the fracture surfaces of the joints were analyzed. The fracture surface in the boundary of the SiC/Sn5Sb3Ti joint is shown in Figure 21a,b. It is obvious that the fracture surface from the side of the SiC ceramic remained covered with solder over approximately 70% of the area. A ductile fracture occurred in the solder. An analysis of the planar distribution of Si, Ti, Ni, Sn and Sb elements on the fracture surface was performed, as shown in Figure 22b–f. From the planar distribution of elements, it is obvious that Ti, Ni and Si elements were segregated on the fracture surface, which enhances the bond formation. This was also proved by the results of the analyses performed on the boundary of the SiC/Sn5Sb3Ti joint, as mentioned in Section 3.5.

**Figure 23:**
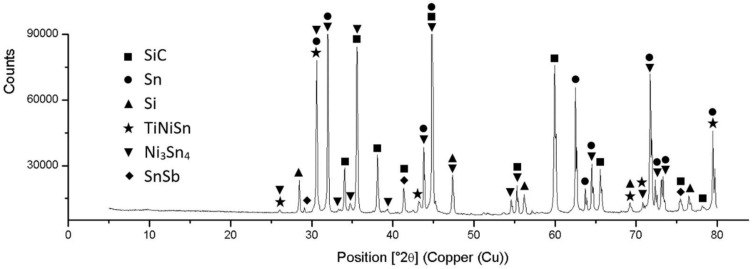
XRD analysis of boundary in SiC/SnSb5Ti3 joint.

An XRD analysis was also performed on the fractured surface of the SiC/Sn5Sb3Ti joint (Figure 23) in order to prove the phases that could be identified on the fractured surface. The titanium phase TiNiSn was thus identified along with the nickel phase Ni_3_Sn_4_, which was also identified by the EDX analysis, and the SnSb phase was also identified, which was proved by the binary diagram Sn-Sb [35].

## 4. Conclusions

The aim of this research was to study the wettability and solderability of SiC ceramics with Ni by the use of a Sn5Sb3Ti solder by heating with an electron beam in a vacuum. The following results were achieved:

- For the determination of the melting points of the solder, the DTA/TG analysis was applied. As a result of DTA analysis, a significant phase reaction with a pronounced thermal effect—a peritectic reaction—was recognized. The onset point at double heating corresponded to the temperatures of 227.8 °C and 225.9 °C, while during cooling it corresponded to 224.6 °C and 224.1 °C.
- The solder matrix was composed of the solid solution (Sn) in which the Sn, Sb and Ti phases were segregated. The presence of a phase with acicular morphology and with high titanium content, Ti_6_(Sn,Sb)_5_, was detected, and the formation of a brittle phase of TiSnSb was observed.
- The results of the solder wettability test on the SiC substrate in a vacuum proved that with increasing temperature the wetting angle decreases. The average wetting angle at 750 °C attained approximately 77°. In the case of the temperature increase at 850 °C, the wetting angle decreased to the average value of 44°. The lowest wetting angle was achieved at the temperature of 950 °C, when it attained an average value around 33°.
- The SiC/solder bond was formed as follows: during the soldering process the titanium from the solder was distributed to the boundary with the ceramic material (SiC) by the mechanism of diffusion. Nickel was dissolved in the tin solder, and it was also distributed to the boundary with the ceramic material by a diffusion mechanism, where a reaction layer ensuring the wetting of the SiC ceramic was formed. The presence of new intermetallic phases was observed: TiSi_2_ and Ti_3_Ni_5_Si_6_, which are the result of the interaction between the solder and the ceramic substrate, and thus the reaction layer was formed.
- A transition zone in the Ni/solder boundary was formed due to the solubility of nickel in tin solder at elevated temperatures. Two new phases, namely Ni_3_Sn_2_ and Ni_3_Sn, were identified in the transition zone with the Ni_3_Sn_2_ phase being around 1 µm in thickness and the zone with the Ni_3_Sn phase being around 10 µm in thickness at the measured point.
- The measurement of shear strength was performed on the joints fabricated at the temperatures of 750, 850 and 950 °C. The highest shear strength was attained at the temperature of 850 °C, around 40 MPa, while the lowest strength was attained at the temperature of 950 °C, around 20 MPa.

## Figures and Tables

**Table 1:** Chemical composition of soldering alloy type Sn-Sb-Ti and the results of chemical analysis performed by ICP-AES method.

Specimen	Charge [wt.%]			ICP-AES [wt.%]		
	Sn	Sb	Ti	Sn	Sb	Ti
Sn5Sb3Ti	92.0	5.0	3.0	balance	5.18 ± 0.26	3.31 ± 0.34

**Table 2:** Soldering parameters.

**Accelerating Voltage**	55.0 kV
**Current**	10.0 mA
**Focusing current**	890.0 mA
**Vacuum**	1 × 10^−2^ Pa
**Heating time**	10.0 min
**Heating temperature**	750 °C, 850 °C, 950 °C
**Time of cooling down**	60 min.
**Distance of jig surface from the electron gun**	200 ± 1 mm

**Table 3:** Parameters of electron beam oscillation.

**Channel A**	13.499 V
**Channel B**	13.499 V
**Frequency**	1000.0 Hz
